# α-Asarone Maintains Protein Homeostasis Through SKN-1-Mediated Proteasome and Autophagy Pathways to Mitigate Aβ-Associated Toxicity in *Caenorhabditis elegans*

**DOI:** 10.3390/antiox14101255

**Published:** 2025-10-18

**Authors:** Congmin Wei, Xinyan Chen, Menglu Sun, Jinjin Cao, Dechun Liao, Zhou Cheng, Hongbing Wang

**Affiliations:** Institute for Regenerative Medicine, Shanghai East Hospital, School of Life Sciences and Technology, Tongji University, Shanghai 200092, China; 1911559@tongji.edu.cn (C.W.); 1434294@tongji.edu.cn (X.C.); sunmenglu@mail.jnmc.edu.cn (M.S.); 1710550@tongji.edu.cn (J.C.); 1632739@tongji.edu.cn (D.L.)

**Keywords:** Alzheimer’s disease, *Acorus tatarinowii* Schott, α-asarone, SKN-1, proteostasis

## Abstract

*Acorus tatarinowii* Schott (*A. tatarinowii*), a traditional Chinese medicine, has been widely used in the treatment of dementia, particularly AD. α-Asarone is the main active component of *A. tatarinowii* oil, and its neuroprotective effects and underlying molecular mechanism in AD remain unclear. In this study, we utilized different transgenic *Caenorhabditis elegans* (*C. elegans*) AD models to investigate the neuroprotective mechanism of α-asarone in vivo. Our findings revealed that α-asarone significantly ameliorated Aβ- and tau-induced phenotypic abnormalities, including deficits in chemotaxis-related learning, hyposensitivity to exogenous serotonin, and impaired neuronal integrity. Furthermore, the α-asarone treatment effectively reduced Aβ-induced oxidative stress. Mechanistically, α-asarone reduced Aβ accumulation and maintained protein homeostasis by stimulating proteasome degradation and autophagy in an SKN-1/Nrf2-dependent manner. Our study highlights the potential of α-asarone as an SKN-1/Nrf2 activator and its capability to facilitate proteostasis, supporting its therapeutic potential for AD treatment.

## 1. Introduction

Alzheimer’s disease (AD) is an aging-related disorder affecting over 52.2 million people worldwide. With the global trend of population aging, AD will not only downgrade life quality for older people but also bring a significant societal financial burden. AD is characterized by amnestic cognitive impairment and neuropsychiatric symptoms. At the pathological level, extracellular amyloid-β (Aβ) plaques and intracellular tau-containing neurofibrillary tangles (NFTs) are the primary hallmarks of AD [1]. Aβ peptides are primarily generated through the proteolytic cleavage of amyloid precursor protein (APP) by β- or γ-secretases; among the various lengths of Aβ fragments, Aβ_40_ and Aβ_42_ are the predominant forms [2]. Although the accurate toxicity mechanisms of Aβ aggregates remain unclear, Aβ oligomerization and fibrilization contribute to neuroinflammation and severe oxidative stress, accelerating AD progression [3]. Over the past few decades, cholinesterase inhibitors such as donepezil, galantamine, tacrine, and rivastigmine have been used to treat mild and moderate AD. Meanwhile, the N-methyl-D-aspartate receptor antagonist memantine is prescribed for severe cases. However, these drugs provide limited benefits and unsatisfactory outcomes. Most of them are also associated with various adverse side effects, including muscle cramps, dizziness, and severe gastrointestinal effects [4]. Over decades of research and drug development efforts, no new therapies emerged, highlighting the unmet need for more disease-modifying interventions that can slow or prevent AD progression.

Proteostasis refers to the maintenance of intercellular protein function and proper concentration through chaperone-mediated protein folding and refolding, the ubiquitin–proteasome system (UPS), and autophagy-associated protein degradation [5]. When proteins failed to degrade, and accumulate abnormally, they will challenge the proteostasis network, disturb protein homeostasis, and ultimately lead to neuronal dysfunction and cell death, driving AD progression [6]. Notably, defective proteostasis is also observed in AD patients, emphasizing the potential therapeutics of targeting and maintaining proteostasis for AD treatment.

The rhizome of *Acorus tatarinowii* Schott (*A. tatarinowii*) is a well-known traditional Chinese medicine frequently used for dementia treatment [7]. α-Asarone (1-propenyl-2,4,5-methoxybenzyl), the major component of *A. tatarinowii*, exhibits various bioactivities, including neuroprotection, antioxidant, anti-inflammatory, and nootropic properties, as well as memory enhancement [8]. However, the underlying neuroprotective mechanism of α-asarone remains controversial.

In this study, we used *Caenorhabditis elegans* (*C. elegans*) as an efficient in vivo model to investigate the neuroprotective effects and mechanisms of α-asarone in AD. *C. elegans* is a well-established model widely used for screening anti-aging and anti-aging-related disease drugs. Additionally, its transparent body and simple and well-characterized nervous system make it an ideal model organism for neurodegenerative disease study [9,10]. This study is the first to determine the potential protective effects and molecular mechanisms of α-asarone using the *C. elegans* model. Our results showed that α-asarone significantly ameliorated Aβ- and tau-induced phenotypic deficit, and enhanced Aβ-induced oxidative stress resistance. Mechanistically, α-asarone activated proteasome and autophagy signaling pathways which protect against Aβ-associated toxicity in an SKN-1-dependent manner. These findings suggested that α-asarone may serve as a potential therapeutic compound for Alzheimer’s disease.

## 2. Materials and Methods

### 2.1. Chemicals and Reagents

α-asarone (98%), Paraquat (Paraquat dichloride hydrate), 5-fluoro-2′-deoxyuridine (FUDR), serotonin, 7′-Dichloro-fluorescein diacetate, diethylpyrocarbonate (DEPC), and Trizol were purchased from Sigma-Aldrich Co., Ltd. (St. Louis, MO, USA). Dimethyl Sulfoxide (DMSO), sodium azide, tunicamycin, and 7-amido-4-methylcoumarin (AMC) were purchased from Sangon Biotech (Shanghai, China) Co., Ltd., while Bortezomib was purchased from MedChemExpress (Monmouth Junction, NJ, USA).

### 2.2. C. elegans Strains

All strains from CGC and *Escherichia coli* (*E. coli*) OP50 were obtained from the Caenorhabditis Genetics Center (University of Minnesota, MN, USA). The PHX3692 strain was generated by SunyBiotech Co., Ltd. using CRISPR/Cas9 system with CL2355 as the background strain. The origin, genotype, and key characteristics of the major C. elegans strains used in this study are listed in Table 1.

### 2.3. C. elegans Maintenance

According to standard protocols, the Bristol CL2006, GMC101, CL2122, CL2355, CL2331, and BC12921 were maintained at 20 °C on nematode growth medium (NGM) agar plates fed with *E. coli* OP50 [11]. These strains, CL4176, CL6180, VH254, VH255, PHX3692, and EG1285 are sensitive to temperature, and were maintained in 16 °C tissue culture incubator.

### 2.4. Paralysis Assay

The synchronized CL4176 L1 larvae were placed on NGM plates containing *E. coli* OP50, treated with or without 20 μΜ α-asarone, after culture at 16 °C for 36 h, and then the culture temperature was shifted to 23 °C for inducing Aβ expression [12]. If the worms failed to move when touched by a platinum pick or a “halo” circle around the anterior area appeared, this represented paralysis.

### 2.5. Chemotaxis Assay

Synchronized CL2355 worm spotting was conducted on NGM culture dishes with 20 μΜ α-asarone or media control until L3 stage, then they were shifted to 25 °C incubator for another 36 h; the worms were collected and washed by M9 buffer (3 g KH_2_PO_4_, 6 g Na_2_HPO_4_, 5 g NaCl, 1 mL MgSO_4_), the drugs were washed out and the worms placed on OP50 plates. A total of 50–60 worms were picked, placed into a chemotaxis plate, attractant (1 M sodium acetate) and control (sterile water) were added at polar ends along with the anesthetic (1 M sodium azide), the worms were treated for 1 h, then numbers on the attractant side and control side were counted; the formula to calculate the chemotaxis index is presented below [13]:

Chemotaxis index = (worms in both test quadrants − worms in both control quadrants)/(total worms)

### 2.6. Serotonin Sensitivity Assay

Synchronized CL2355 worms were fed with or without 20 μΜ α-asarone on NGM culture agar dishes at 16 °C until adult, shifted to 25 °C for another 36 hrs, worms were collected, at least sixty worms in each group, washed with M9 buffer for three times, then transferred into 96 wells plate containing 200 μL 5 mg/mL serotonin (dissolved in M9); we put 10 worms per well, and counted the active worms after 12 h-treatment.

### 2.7. Amyloid-Peptide Deposition Assay

The CL2331 transgenic strain was used to test Aβ deposit in worms. Briefly, synchronized CL2331 animals were exposed to α-asarone (20 μΜ) or dmso control, 200 μM FUDR was added to block progeny development at the L3 stage. Nematodes were cultured at 20 °C (to induce Aβ expression) until day 2 of adulthood, then the worms were collected. Fluorescence intensity was detected by fluorescence microscope. The fluorescence intensity was quantified by Image-J 1.52a software.

### 2.8. Oxidative Stress Assay

Oxidative stress was induced by paraquat. Synchronized L1 larvae (CL2006) were treated with 20 μΜ α-asarone or solvent control, then incubated in liquid S-complete with *E. coli* OP50 bacteria at 20 °C for 4 days. During this time, FUDR (1.08 mM) was added to block progeny development at the L3 stage. Subsequently, 4-day adult worms were exposed to 50 mM paraquat and the survival number was scored each hour until all worms died.

### 2.9. ROS Accumulation Assay

Synchronized CL4176 worms were treated with α-asarone (20 μΜ) or control. Worms were collected after culture at 23 °C for 32 h, then treated with 50 μM 2′,7′-Dichloro-fluorescein diacetate (H_2_DCF-DA; Molecular Probes (Waltham, MA, USA)), incubated at 37 °C for another 2 h to label the intracellular reactive oxygen species (ROS). Subsequently, quantification of fluorescence was measured in SpectraMax M5 Fluorescence Microplate Reader (Molecule Devices, San Jose, CA, USA), samples were read every 20 min for 2 h; the excitation was 485 nm, and the emission was 535 nm.

### 2.10. Development Arrest Assay

Worms were treated with bortezomib which dysregulates proteasome-related activities and prevents larvae from developing to adult. Synchronized CL2122, GMC101, and CL6180 L1 larvae were cultured in 96-well plates. Each well of a 96-well plate was set up with a total volume of 150 µL containing 15 µL of 4 µg/mL bortezomib (final concentration: 0.4 µg/mL), 10–15 synchronized worms, 10 µL OP50 and 15 µL 200 μΜ α-asarone (final concentration: 20 μΜ), the remaining volume was made up with S-complete medium, administered for 3 days. The diapause portion was calculated.

### 2.11. Proteasome Activity Assay

Chymotrypsin-like activity could mimic proteasome activity; we detected the chymotrypsin-like activity by using Suc-LLVY-AMC fluorescent kit, which was purchased from Sigma-Aldrich (St. Louis, MO, USA). A total of 1000 synchronized CL4176, CL6180, and CL802 worms had been used in this assay, synchronized worms were treated with or without 20 μΜ α-asarone, worms were let to grow up at 16 °C for 36 h, then raised to 23 °C for another 32 h. The worms were collected and lysed with proteasome activity assay buffer (5 mM ATP, 10 mM DTT, 0.5 mg/mL BSA, 5 mM MgCl_2_, 40 mM KCl, 1 M Tris-HCl, pH 7.5) on ice for 5 min. The lysate was centrifuged at 10,000× *g* for 15 min at 4 °C, the total protein was quantitated by BCA Kit (Thermo Fisher Scientific, Waltham, MA, USA) and adjusted to 5 μg/mL for each sample. A total of 50 μL protein samples, 100 μL 200 μM Suc-LLVY-AMC and 50 μL proteasome activity assay buffer were added to a 96-well plate. Fluorescence was recorded with the iD5 microplate reader every 5 min for 150 min at 37 °C; the excitation wavelength was 380 nm, and the emission wavelength was 460 nm.

### 2.12. Measuring the P62 Protein Fluorescence Intensity in BC12921 Worms

Worms were treated with or without 20 μΜ α-asarone for 5 days, after that, the worms were collected in a black 96-well plate containing 100 μL M9 buffer per well, 15 worms were put in each well. P62 protein fluorescence intensities were measured with SpectraMax M5 fluorescence microplate reader; excitation and emission wavelengths were 485 and 535 nm. This assay was replicated more than two times, a statistical significance test Student’s *t*-test was conducted, and the *p*-value was defined as *p* < 0.05 [14].

### 2.13. Thrashing Assay

Synchronized VH255 (wildtype(wt) tau transgenic worms) and VH254 (pseudo hyperphosphorylated (PHP) tau transgenic worms) worms were cultured in 96 wells (15 worms per well in 110 μL of S-Complete/OP50 mixture) at 20 °C until they reached the L3 larval stage. Then, 25 μL of 1.08 mM FUDR was added to each well to inhibit egg-laying. After the worms developed to the adult stage, 15 μL of the α-asarone was added to each well until the Day 2 adult stage was reached; thrash frequency was recorded in 10 s using Wormlab^®^ Imaging system from MBF Bioscience (Williston, VT, USA).

### 2.14. RNA Extracted and Quantitative RT-PCR

The synchronized CL4176 L1 larvae were placed on NGM plates, fed with *E. coli* OP50, supplemented with or without 20 μΜ α-asarone at 16 °C for 36 h, then raised to 23 °C for another 32 h. Worms were collected and washed with diethylpyrocarbonate (DEPC) water, RNA was extracted by Trizol, isolated RNA was stored at -80 °C for further use. Complementary DNA (cDNA) was prepared by using a first-strand synthesis system for Quantitative real-time PCR (qRT-PCR). qRT-PCR was performed by using 2× SYBR Green qPCR Master Mix (Bimake, Shanghai, China) and tested with LC96 real-time PCR detection system.

The below qRT-PCR reaction conditions were followed:

95 °C 5 min, followed by 40 cycles of 15 s at 95 °C, and 45 s at 60 °C.

The primers are summarized in Supplementary Table S11; *actin-1* was used as an internal control, and relative fold-change for transcripts were analyzed by the 2^−ΔΔCt^ method [15]. The relative expression of target genes was calculated using the 2^−ΔΔCt^ method. Briefly, the Ct value of each gene was first normalized to a stable housekeeping gene (ΔCt), with GAPDH used as the housekeeping gene in this study. The ΔCt of α-asarone-treated samples was then compared to the average ΔCt of untreated samples (ΔΔCt). The fold change in gene expression was calculated as 2^−ΔΔCt^, representing the expression level of each gene relative to the control group.

### 2.15. Western Blotting Assay

The CL4176 worms were treated with or without 20 μΜ α-asarone for 32 h after shifting the temperature to 23 °C, harvested in PBS buffer, sonicated in RIPA buffer with 1× protease inhibitor cocktail (87786, Thermo); the protein was quantified with BCA kit (23227, Thermo). Before loading, the samples were boiled in sample buffer (P1015, Solarbio (Beijing, China)) for 3 min. The target proteins were identified by the Tris-Tricine gel [12], 60 μg total in each lane was loaded, the gel was run at 80 V for 4–6 h on ice or 4 °C. After running, the samples were transferred to 0.2 μΜ PVDF membrane; the transfer conditions were 12 V, 17 min. The membrane was blocked in skim milk for 2 h. The membrane was incubated overnight at 4 °C with these primary antibodies: mouse anti-Aβ (6E10, Biolegend (San Diego, CA, USA)), mouse anti-proteasome 20S alpha 1_7 (MCP231, Santa Cruz (Dallas, TX, USA)), mouse anti-rpn-10 (398033, Santa Cruz), mouse anti-rpt-6 (PW8320, Enzo Life (Farmingdale, NY, USA)), mouse anti-beta-actin (60008, Proteintech (Rosemont, IL, USA)), mouse anti-gapdh (60004, Proteintech), rabbit anti-nrf2 (16396, Proteinthch). The next day, the membrane was washed three times with TBS-T, 10 min each time, the membrane was incubated with secondary antibodies at room temperature for 1 h: anti-mouse IgG-HRP (7076, Cell Signaling Technology (Danvers, MA, USA)) and Anti-Rabbit IgG (ab288151, Abcam (Waltham, MA, USA)). After incubation, the membrane was washed three times with TBS-T, 10 min each time, developed with GE ECL detection system (Beyotime, Shanghai, China).

### 2.16. RNAi-Mediated Interference (RNAi) Assay

The synchronized CL4176 were cultured on NGM plates containing 1 mM isopropyl-β-D-1-thiogalactopyranoside (IPTG) (Sangon (Shanghai, China)) and seeded with HT115 cloned target gene (bec-1_F:GCTCTAGAATGACGACCCAACGAAGCCA,bec-1_R: GGGGTACCCTAAATAGGCGATCTGA GAG) or empty L4440 vector. The bec-1 gene is the *C. elegans* ortholog of mammalian Beclin-1. It is essential for initiating autophagosome formation. Inhibition of bec-1 consistently blocks autophagy, leading to increased protein aggregation and stress sensitivity in worm models. Cultured on RNAi NGM plates for two generations. The RNAi interference worms strain was named as CL4176; bec-1(-/-), and paralysis assay with this strain was performed.

### 2.17. Neuron Degeneration Assay

For analysis of the development of neurons in Aβ expressing worms, we generated a new transgenic strain PHX3692 under the CL2355 strain. mCherry and GABA vesicular transport expression were driven by *unc-47* promoter [16]. The DNA plasmid mixture containing 20 ng/μL pPD95.77 (Punc-47::mCherry) and 50 ng/μL report marker (*rol-6*(su1006)) were injected into the gonads of adult CL2355 hermaphrodite nematodes by using a microinjection machine [17]. This experiment was commissioned by SunyBiotech.

The neurodegeneration assay was performed with this new PHX3692 strain. Synchronized L1 larvae were fed with or without α-asarone, the worms were collected at the L4 stage, worms were arranged on the agarose pad, the worms’ ventral nerve cord D-type neurons were observed by using a Leica SP8 (Leica Microsystems (Wetzlar, Germany)) confocal under × 63 oil objectives. In general, there are 21 D-type neurons in worms, and Aβ expression damages neuron development and might reduce the D-type neuron number; in this experiment, we recorded worm numbers with D-type neurons less than 16 [18].

### 2.18. Statistical Analysis

All quantified data are presented as Mean ± SD. Statistical differences were determined by one-way ANOVA. If the ANOVA was significant, the data were analyzed by Student’s *t*-test to determine statistical significance with GraphPad Prism 6.0 software (GraphPad, La Jolla, CA, USA). *p* < 0.05 was considered statistically significant.

## 3. Results

### 3.1. α-Asarone Ameliorated Aβ and Tau-Induced Damage in C. elegans

**Figure 1:**
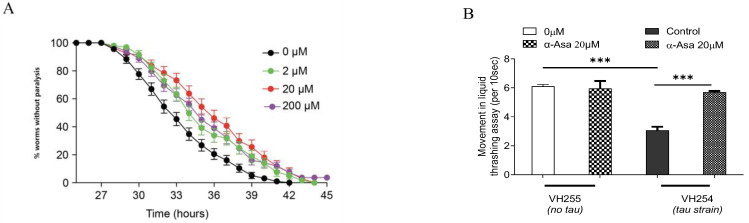
Effects of α-Asarone on Aβ-induced paralysis and tau-induced damage in nematodes. (**A**) α-Asarone delayed Aβ-induced paralysis in CL4176. The assays were tested three times, and there were at least 30 worms for each assay. Statistical analysis is shown in Table S1. (**B**) Liquid thrashing assay in tau transgenic worms. VH255 is the control strain for VH254 which expresses pseudohyperphosphorylated (PHP) tau; the thrashing rate of each worm for 10 s was recorded in liquid, the assays were tested three times., and there were 30 worms for each assay. Statistical analysis is shown in Table S2. The error bar presents mean ± SD, “***” represents *p* value < 0.001.

We first examined the protective effects of α-asarone against Aβ-induced toxicity in vivo using the Aβ transgenic CL4176 strain. This strain expresseses human Aβ peptide in its muscle cells in a temperature-sensitive way. At the L3 larval stage, shifting the culture temperature from 16 °C to 23 °C induces Aβ expression and accumulation in muscle cells. Excessive Aβ accumulation leads to cellular toxicity, manifesting as a progressive paralysis phenotype. In our study, we treated CL4176 worms with α-asarone, up to 200 μM, with no observed survival toxicity. The paralysis assays were quantified for mean time duration at which 50% worms were paralyzed (PT50) from the transgenic worms fed with or without the drugs [12]. Paralysis-based analysis indicated that 20 μM α-asarone treatment showed the most effective protective effect, significantly increasing the PT_50_ to 9.73 ± 0.32 h, a 62.17% improvement compared to the control (6.00 ± 0.20 h) (Figure 1A, Table S1).

Tau, a predominant microtubule-associated protein, is expressed less in neuronal axons and is critical in maintaining neuronal function. In AD progression, Aβ deposition and senile plaque formation elicit various secondary effects, including facilitating the development of hyperphosphorylated tau, to cause neuronal dysfunction and synaptic damage [19]. Our study demonstrated that α-asarone reduced Aβ accumulation-induced damage in *C. elegans*. To assess its impact on tau-induced toxicity, we performed a liquid thrashing assay using the tau transgenic strains. The VH254 strain pan-neuronally expresses pseudo-hyperphosphorylated tau—a pathological form commonly found in AD. And the VH255 strain, expressing wild-type human tau, served as a control [20]. Worms expressing PHP tau exhibit neuromuscular defects, including impaired crawling and a reduced thrashing rate. Treatment with 20 μM α-asarone significantly improved the motility (Figure 1B), increasing the 10 s average thrashing rate in liquid to 5.705 ± 0.0619 thrashes, an 83.9% improvement compared to the untreated control group (3.198 ± 0.1016) (Table S2), suggesting that α-asarone mitigates PHP tau-induced motor deficits. In parallel, 20 μM α-asarone did not affect the thrashing rate of VH255 wild-type worms, implying that its protective role is specific to pathological tau (Figure 1B, Table S2). Our data showed that α-asarone ameliorated Aβ-induced neurotoxicity and also improved PHP tau-associated motility dysfunction in *C. elegans*, highlighting its potential as a therapeutic candidate for AD.

### 3.2. α-Asarone Mitigates Aβ-Induced Neurotoxicity by Enhancing Chemotaxis, Serotonin Sensitivity, and GABAergic Neuron Integrity in C. elegans

**Figure 2:**
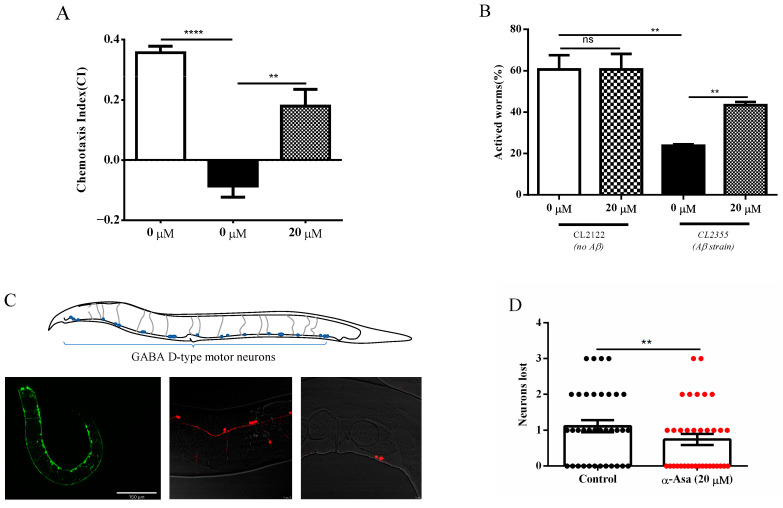
The neuroprotective effects of α-asarone in worms expressing Aβ in pan-neuron. (**A**) α-asarone improved chemotaxis in CL2355 nematodes. The CI is 0.18 ± 0.03 in the α-asarone group, and −0.08 ± 0.02 in the control group; there were at least 150 worms in total. Statistical analysis is shown in Table S3, error bars indicate SD, ** *p* < 0.01, **** *p* < 0.0001. (**B**) α-asarone enhanced 5-HT sensitivity in CL2355. The percentage of active worms is 43.38 ± 1.06%, there were at least 90 worms in total. Statistical analysis is shown in Table S4, error bars indicate SD, ** *p* < 0.01. (**C**) Representative images of the ventral nerve cord D-type neurons in EG1285 and PHX3692 nematodes. The upper image is the schematic drawing of the positions of the 19 GABA D-type motor neurons in *C. elegans*, the bottom left is a representative image of D-type motor neurons in the EG1285 animal which is the control strain of PHX3692 (ECHO Revolve Microscope, scale bar is 150 μm), bottom middle is a representative image of D-type neurons in PHX3692 (Leica SP8 Microscope, scale bar is 10 μm), bottom right is a representative image of worm-exhibited D-type neuron loss (Leica SP8 Microscope, scale bar is 10 μm). (**D**) α-asarone treatment reduced the number of worms that lost neurons in PHX3692 animals compared with the control group; there were at least 30 worms in total. Mean ± SD is shown, ** *p* < 0.01.

Amyloid peptide accumulation in neurons impairs olfactory-related learning capabilities in nematodes [12]. Our primary finding indicated the protective effects of α-asarone in the context of Aβ and tau aggregates. To further access the neuroprotective effects of α-asarone, we performed a chemotaxis behavior assay using the pan-neuronally expressing transgenic strain CL2355. Perceiving chemical signals and adapting to environmental changes are critical for *C. elegans* survival. Sodium acetate, a natural attractant from food bacteria, generally induces chemotaxis in *C. elegans*. However, Aβ deposition affects the neuron system, impairing chemotactic response. The worms were placed on assay plates containing sodium acetate for 60 min. We observed that treatment with 20 μM α-asarone significantly enhanced chemotactic behavior in CL2355 worms. The chemotaxis index (CI), defined as the fraction of worms gathered in the chemotaxis attractant area [13], was significantly higher in the α-asarone treatment group (CI = 0.18 ± 0.03) compared to the control group (CI = −0.08 ± 0.02) (Figure 2A, Table S3). These results suggest that α-asarone mitigates Aβ-induced deficits in chemotactic behavior.

5-hydroxytryptamine(5-HT), a crucial neurotransmitter, modulates various behaviors in *C. elegans*, including locomotion, pharyngeal pumping, and egg-laying [21,22]. In general, *C. elegans* is sensitive to excessive 5-HT, and exposure to exogenous 5-HT leads to paralysis. To further confirm the neuroprotective effect of α-asarone, we tested worm response activity to exogenous 5-HT. In this study, 5-HT treatment decreased the population of active worms in the CL2355 strain compared to its control strain CL2122 (Figure 2B). It was suggested that Aβ expression impaired the worm’s sensitivity to exogenous 5-HT. Interestingly, α-asarone treatment significantly reduced worms’ paralysis. The population of active worms is 43.38 ± 1.06%, compared to only 23.75 ± 0.49% in the control group (Figure 2B, Table S4).

To assess the protective effects of α-asarone in neuro integrity, we established the transgenic *C. elegans* reporter strain PHX3692 by fusing mCherry fluorescent protein with gamma-aminobutyric acid (GABA), specifically expressed in the GABA D-type neuron (Figure 2C). This strain enables the in vivo assessment of GABAergic motor neuron integrity along the ventral nerve cord. Confocal microscopy images revealed that the PHX3692 strain exhibited significantly incomplete D-type neurons compared to the control strain EG1285 (Figure 2C). Additionally, the α-asarone treatment group showed less neuronal loss than the untreated group. In this experiment, neuronal dysfunction was defined as the loss of more than two D-type neurons. Based on this criterion, the number of nematodes losing GABA D-type motor neurons was significantly lower in the α-asarone-treated group compared to the untreated group (Figure 2D). These results suggest that α-asarone enhances chemotaxis, improves sensitivity to exogenous serotonin, and preserves neuronal integrity in the ventral nerve cord, thereby alleviating Aβ aggregation-induced neurotoxicity in vivo.

### 3.3. α-Asarone Decreased Amyloid Peptide Deposition in Nematodes

**Figure 3:**
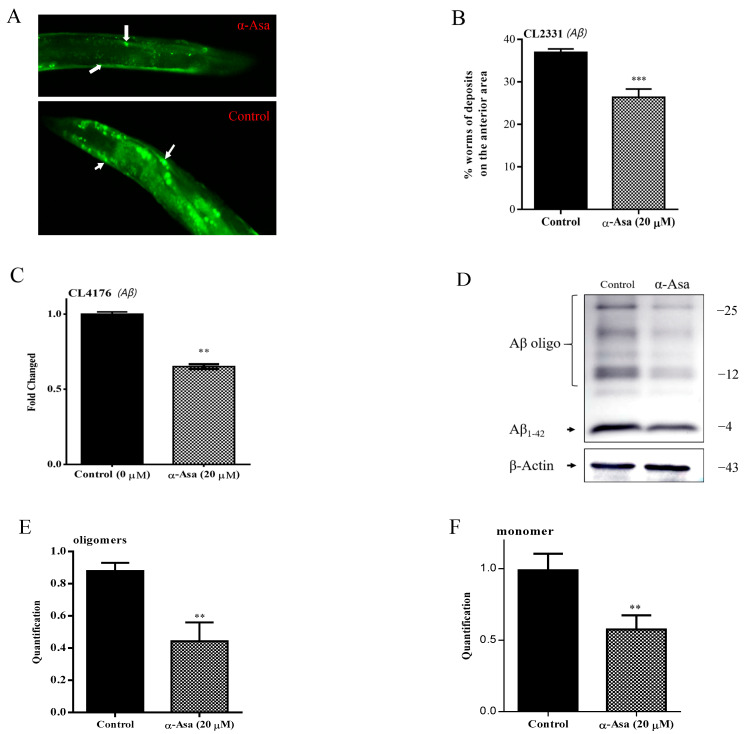
α-asarone decreased transcription and protein levels of Aβ in AD worms. (**A**) The representative fluorescence images in the anterior area in CL2331 worms, arrows indicate the accumulation of Aβ amyloid protein. (**B**) Statistics of the relative fluorescence intensity in the anterior area treated with 20 μΜ α-asarone or dmso control; the assays were tested three times., and there were 1000 worms in total. Fluorescence intensity analysis is shown in Table S5. (**C**) Aβ mRNA level in CL4176 worms treated with dmso control or α-asarone for 32 h after shifting the culture temperature to 23 °C; the assays were tested three times, and there were 1000 worms in total. (**D**) Aβ protein levels in CL4176 nematodes. The Western blotting assay was used to analyze the amount of Aβ monomers and oligomers, β-Actin is the internal reference. (**E**) Quantitative analysis of Aβ oligomers in each group. (**F**) Quantitative analysis of monomers in each group. The Error bar presents mean ± SD, ** *p* < 0.01; *** *p* < 0.001.

In mammalian systems, Aβ tends to aggregate extracellularly, forming oligomers and fibrils that contribute to synaptic dysfunction and neuronal death [23]. Our results indicated that α-asarone preserves neuronal integrity and alleviates paralysis caused by Aβ expression in nematodes. To further investigate how access to α-asarone influences Aβ deposition and associated proteotoxicity in vivo, we employed the CL2331 *C. elegans* strain, in which Aβ is expressed intracellularly in the body wall muscle cells and forms aggregates in the anterior pharyngeal region [12]. The fluorescence images revealed substantial mainly amyloid deposits across the anterior area (Figure 3A). Quantitative analysis of relative fluorescence intensity analysis showed that α-asarone treatment significantly reduced amyloid deposition by 28.6% (26.39 ± 1.12%) compared to the control group (36.96 ± 0.45%) (Figure 3B, Table S5).

Additionally, we also examined the transcription and protein levels of Aβ. The results showed that 20 μΜ α-asarone significantly reduced the Aβ mRNA levels (Figure 3C). Immunoblotting analysis revealed multiple Aβ oligomers species in worms in addition to Aβ monomer (Figure 3D). Consistent with the transcription data, α-asarone treatment significantly decreased both Aβ monomer (~4 KDa) and Aβ oligomers expression levels (Figure 3E,F). These findings suggested that α-asarone mitigates Aβ-induced damage by reducing both Aβ transcription and protein levels in nematodes.

### 3.4. α-Asarone Attenuates Aβ-Induced Oxidative Stress by Enhancing Antioxidant Defense and Stress Resistance in C. elegans

**Figure 4:**
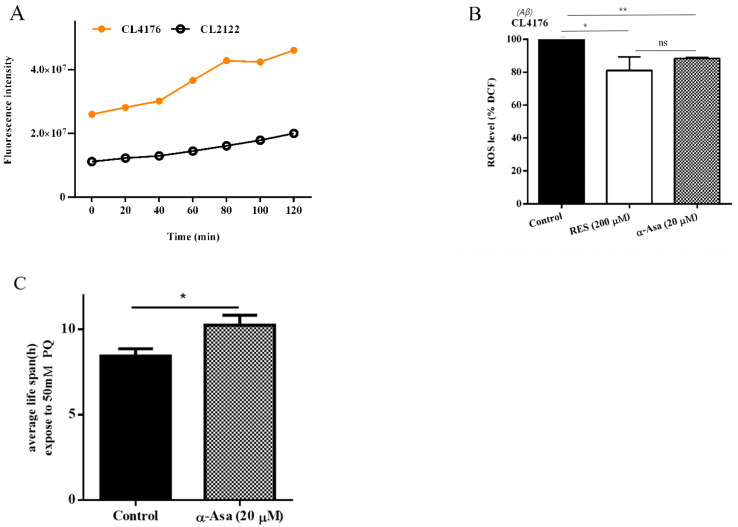
Protective effect of α-asarone on oxidative stress resistance in nematodes. (**A**) ROS accumulation in the CL4176 strain and CL2122 control strain. (**B**) ROS levels in CL4176 nematodes. Resveratrol (RES) is the positive control in this assay, the ROS level of the control group was set as 100%, data are presented in Table S6. (**C**) The average lifespan of worms exposed to 50 mΜ paraquat. Statistical analysis is presented in Table S7; all these experiments were repeated three times (or more than 60 worms total). Value is presented as mean ± SD. ns *p* > 0.05, * *p* < 0.05, ** *p* < 0.01.

Oxidative stress plays an important role in the progression of neurodegenerative diseases [24], and clinical studies have reported elevated ROS levels in AD patients [25]. Previous in vivo and in vitro studies have demonstrated the antioxidant capacity of α-asarone. To determine whether the neuroprotective effects of α-asarone are mediated through antioxidant capacity, we conducted an H_2_DCF-DA probe-based ROS assay. We observed that the CL4176 strain exhibited significantly higher ROS levels compared to the non-Aβ expressing control strain CL2122 (Figure 4A). In this study, resveratrol was used as a positive control due to its well-documented antioxidative properties and ROS-scavenging capability in vivo [26]. Treatment with 20 μM α-asarone reduced ROS levels in CL4176 worms to 86.79 ± 1.64%, comparable to the 81.28 ± 3.31% observed in worms treated with resveratrol (Figure 4B, Table S6), suggesting that α-asarone exerts a potent antioxidant effect at a lower dosage.

Paraquat, a rapid-contact herbicide, induces oxidative stress by increasing the production of superoxide anions [27]. To further validate the antioxidant potential of α-asarone in vivo, we performed a paraquat-induced oxidative stress resistance assay. Exposure to paraquat significantly reduced the lifespan of C. elegans; however, α-asarone treatment ameliorated paraquat-induced toxicity, increasing the average lifespan by 21.47% compared to the control group (control: 8.43 ± 0.31 h, α-asarone: 10.24 ± 0.34 h; Figure 4C and Table S7). These findings confirmed the antioxidant properties of α-asarone and suggested that α-asarone protects against Aβ-induced neurotoxicity by enhancing oxidative stress resistance.

### 3.5. α-Asarone Enhances Oxidative Stress Resistance via SKN-1/Nrf2 Pathway Activation to Protect Against Aβ Toxicity in C. elegans

**Figure 5:**
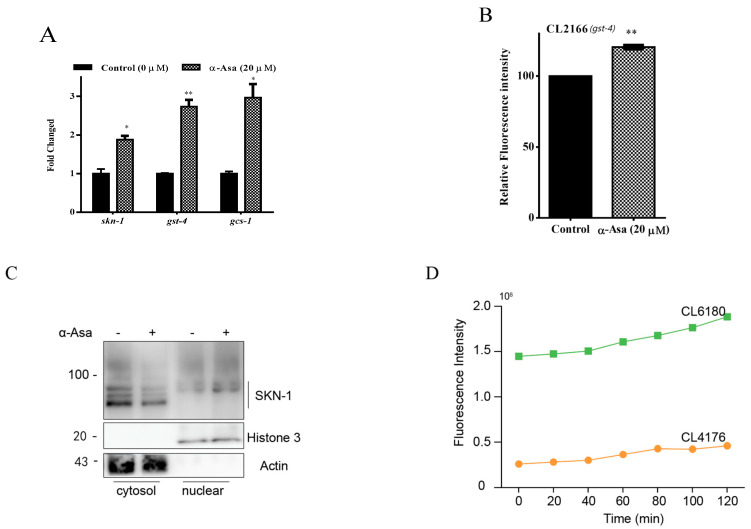

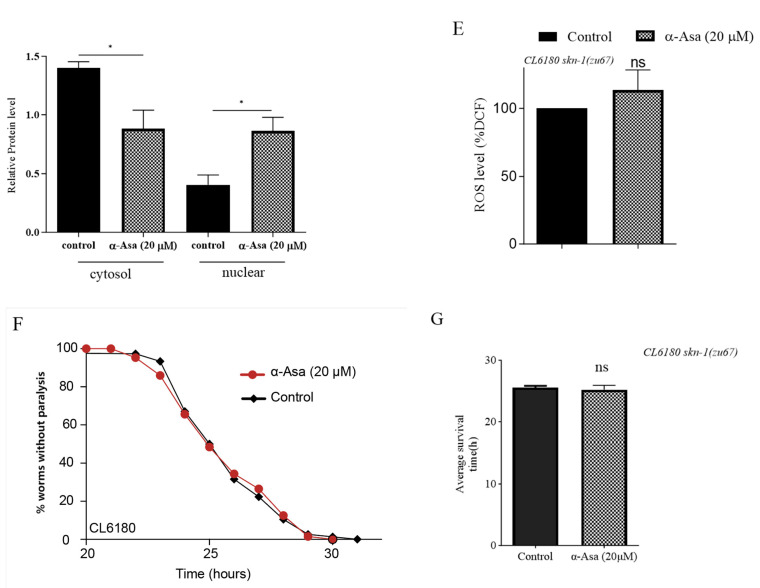
α-asarone mediated neuroprotection requires the regulation of SKN-1. (**A**) The transcription levels of *skn-1* and its target genes in CL4176 nematodes; the assays were tested three times, and there were 1000 worms in total. (**B**) The relative fluorescence intensity of GST-4 in the CL2166([(pAF15) gst-4p::GFP::NLS] III) strain was quantified by ImageJ; there were 90 worms in total. Detailed data is shown in Table S8. (**C**) Western blotting analysis of cytosol and nuclear Nrf2 protein levels in CL4176 nematodes; the assays were tested three times, and there were 1000 worms in total. (**D**) The ROS accumulation levels in CL4176 and CL6180 nematodes (more than 60 worms in total). (**E**) The ROS levels of CL6180 worms after being fed with α-asarone and DMSO control. There is no significant difference between the α-asarone group and the control group; the assays were tested three times., and there were 30 worms for each assay. Analysis data is presented in Table S9. (**F**) Curves of worms not paralyzed in the CL6180 mutant strain. (**G**) The average lifetime of CL6180 in the α-asarone and control group (more than 60 worms in total). Value is presented as mean ± SEM, ns *p* > 0.05, * *p* < 0.05, ** *p* < 0.01.

Transcription factor SKN-1, a homolog of mammalian Nuclear Factor E2-Related Factors (Nrf2), is typically maintained at a low steady level due to rapid degradation by the ubiquitin–proteasome system (UPS) [28]. However, excessive oxidative stress and disruption of UPS result in SKN-1 stabilizing and translocating into the nucleus, where it binds to specific DNA regions (antioxidant response elements, AREs) to activate oxidative stress defense mechanisms [29]. Based on our findings (Figure 4B), we hypothesized that the protective properties of α-asarone are mediated through the SKN-1 pathway. To test this, we examined the transcription levels of *skn-1* and its downstream target genes *gst-4* and *gcs-1*. Our results showed that treatment with α-asarone significantly upregulated *skn-1* and *gst-4*, *gcs-1* mRNA levels (Figure 5A). Additionally, we also assessed GST-4 expression in CL2166 strains, which express GST-4 fused with GFP, and observed that α-asarone treatment increased GST-4 expression (Figure 5B, Table S8). Moreover, the immunoblotting results further confirmed that α-asarone treatment promoted SKN-1 translocation to the nucleus, indicating that α-asarone activates the SKN-1-mediated antioxidative pathway (Figure 5C).

To elucidate the role of SKN-1 in the neuroprotective effects of α-asarone, we investigated its protective effects in an SKN-1 deficient background using the skn-1 mutant Aβ transgenic CL6180 strain. Compared to CL4176, SKN-1 deficient nematodes exhibit excessive ROS accumulation (Figure 5D). Notably, treatment with α-asarone failed to reduce the abnormally high ROS levels in CL6180 nematodes (Figure 5E). Similarly, α-asarone treatment did not mitigate Aβ-expression-induced paralysis in the *skn-1* mutant CL6180 strain (Figure 5F,G, Table S9). All these results initially suggested that SKN-1 is a key regulator of the antioxidative and neuroprotective effects of α-asarone against Aβ-induced toxicity.

### 3.6. α-Asarone-Decreased Aβ Accumulation Relies on Proteasome-Related Degradation Pathway

**Figure 6:**
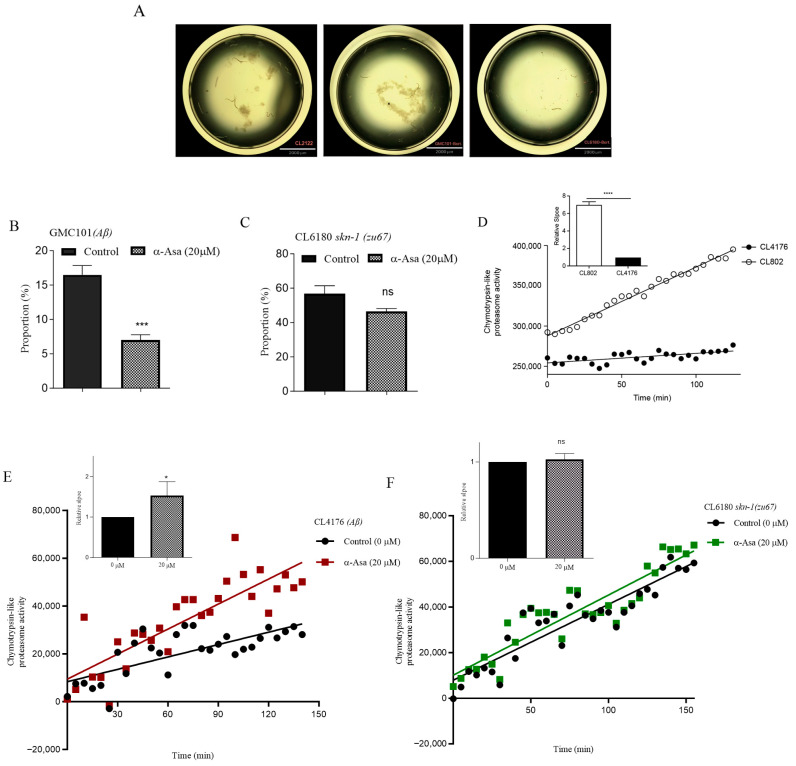
α-asarone retarded Aβ accumulation-induced damage through SKN-1-mediated proteasome activity. (**A**) Representative images for developmental arrest in different strains. **Left**: CL2122, **middle**: GMC101, **right**: CL6180; the abbreviation “Bort” denotes bortezomib, all these images were captured at the stage resembling the CL2122 L4 stage, scale bar is 2000 μm under the microscope. (**B**) α-Asarone delayed bortezomib-induced diapause in GMC101 nematodes. Untreated group: 16.49 ± 0.78%; treated group: 7.00 ± 0.44%. (**C**) α-Asarone did not affect the diapause induced by bortezomib in *skn-1* mutant CL6180 nematodes. Untreated group: 56.84 ± 4.59%; treated group: 46.38 ± 1.79%. (**D**) Amyloid accumulation decreased the proteasome activity in nematodes. The histogram is the relative slope of each assay; scatter diagram: the relative fluorescence units per minute (RFU min^−1^) are calculated using the linear slope method [32]. (**E**) α-Asarone enhanced the proteasome activity in CL4176 worms. (**F**) α-Asarone showed no effect on the proteasome activity in *skn-1* mutant CL6180 strains. All these experiments were repeated three times, and all these experiments have at least 30 worms for each treatment, error bar: mean ± SEM. ns *p* > 0.05, * *p* < 0.05, *** *p* < 0.001, **** *p* < 0.0001.

SKN-1 is not only a master regulator of oxidative stress but also plays a critical role in maintaining protein homeostasis by regulating proteasome function [30]. Our findings indicated that α-asarone enhanced oxidative stress resistance in *C. elegans* mediated by SKN-1. Whether this process also strengthens SKN-1-associated UPS function remains unclear. To investigate this, we performed a proteasome activity assay using the Aβ transgenic GMC101 strain in liquid culture, with bortezomib as a proteasome inhibitor. Bortezomib reversibly binds to the β5-subunit of the 20S proteasome, inhibiting chymotrypsin-like activity and leading to proteostasis dysfunction [31]. Importantly, 16.49% larvae were arrested at the L1 or late L3 stage in the GMC101 strain, with no observed developmental arrestment in the CL2122 control stain, suggesting that Aβ expression accelerates bortezomib-induced proteosome inhabitation (Figure 6A,B). Meanwhile, α-asarone treatment significantly reduced the incidence of developmental arrest to 7% in GMC101 worms (Figure 6B). Furthermore, we observed severe diapause symptoms in the *skn-1* mutant CL6180 strain, with 56.84% larvae arrested at the L1 stage or dying. Notably, α-asarone treatment failed to rescue this phenotype in CL6180 worms (Figure 6C).

The ubiquitin–proteasome system is responsible for degrading intracellular proteins properly to maintain protein homeostasis [31]. Excessive Aβ aggregation disrupts UPS function and threatens proteostasis. Bortezomib-induced proteasome inhibition impaired larval development, while α-asarone alleviated this defect, suggesting that α-asarone may restore chymotrypsin-like proteasome activity. To confirm this, we measured proteasome activity in nematodes using fluorogenic peptide substrates. As expected, Aβ expression significantly reduced proteasome activity in CL4176 worms compared to non-Aβ-expressing CL802 worms (Figure 6D). However, α-asarone treatment markedly restored proteasome activity in CL4176 worms (Figure 6E). In contrast, α-asarone failed to rescue the proteasome activity deficit in the *skn-1* mutant CL6180 strain (Figure 6F). These results further confirmed that SKN-1 is a key regulator in mediating the protective effects of α-asarone.

**Figure 7:**
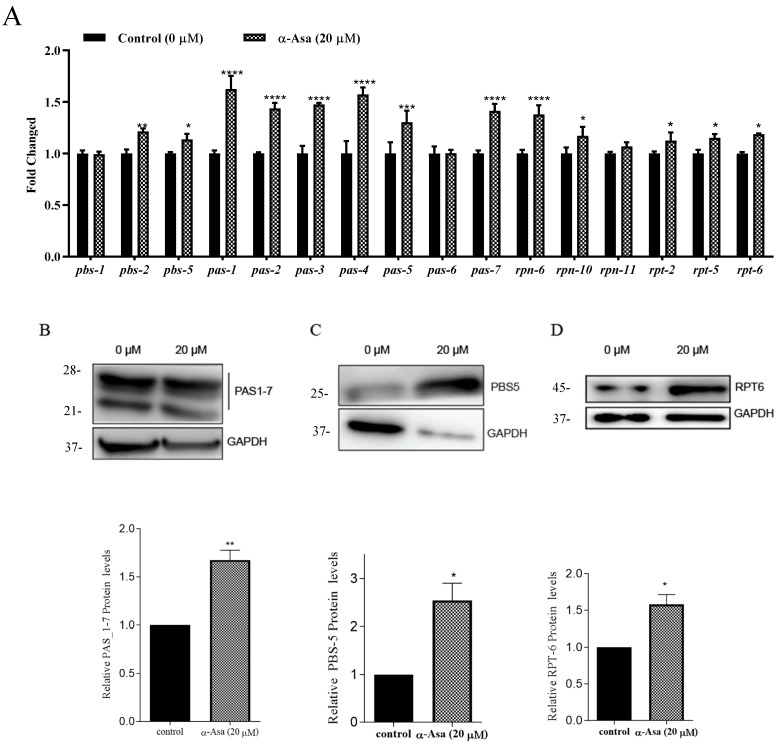
α-asarone upregulated the expression levels of proteasomal genes to improve proteasome abundance in nematodes. (**A**) The transcription levels of proteasomal subunits in the CL4176 strain. (**B**) α-Asarone increased the protein levels of 20S proteasome α-subunit_1-7(PAS_1-7) in CL4176 strains. GAPDH as an internal control. **Top**: the immunoblotting image; **bottom**: quantification for PAS expression in α-asarone treated and untreated groups. (**C**) The protein levels of proteasome β-subunit 5 (PBS-5) were increased in the 20 µM α-asarone treatment group compared to the untreated group. (**D**) α-Asarone increased the protein levels of RPT-6 in CL4176 nematodes. All these experiments were repeated three times (and more than 1000 worms in total). Error bars represent mean ± SD, * *p* < 0.05, ** *p* < 0.01, *** *p* < 0.001, **** *p* < 0.0001.

Given that α-asarone affected proteasome activity to protect against Aβ-induced injuries in *C. elegans*, we evaluated the expression levels of proteasomal subunits. QPCR results showed that α-asarone treatment significantly upregulated the transcription levels of genes encoding proteasomal α and β subunits, including *pas-1,2,3,4,5,7* (which encode α-rings of 20S proteasome) as well as *pbs-2*, *pbs-5* (which encode the β-rings of 20S proteasome). Additionally, α-asarone increased the mRNA levels of *rpn-6*, *rpn-10*, *rpt-2*, *rpt-5* and *rpt-6*, which encode components of 19S regulatory subunits [33] (Figure 7A). Consistent with these mRNA expression results, α-asarone treatment significantly increased the protein levels of PAS1_7, PBS-5, and RPT-6 (Figure 7B–D).

Taken together, all these results suggested that α-asarone enhances proteasome abundance to facilitate proteostasis against Aβ-induced damage. Furthermore, these protective effects appear to be SKN-1 dependent.

### 3.7. α-Asarone-Retarded Aβ Accumulation Likely Relies upon the Autophagy-Lysosome Protein Degradation Pathway

**Figure 8:**
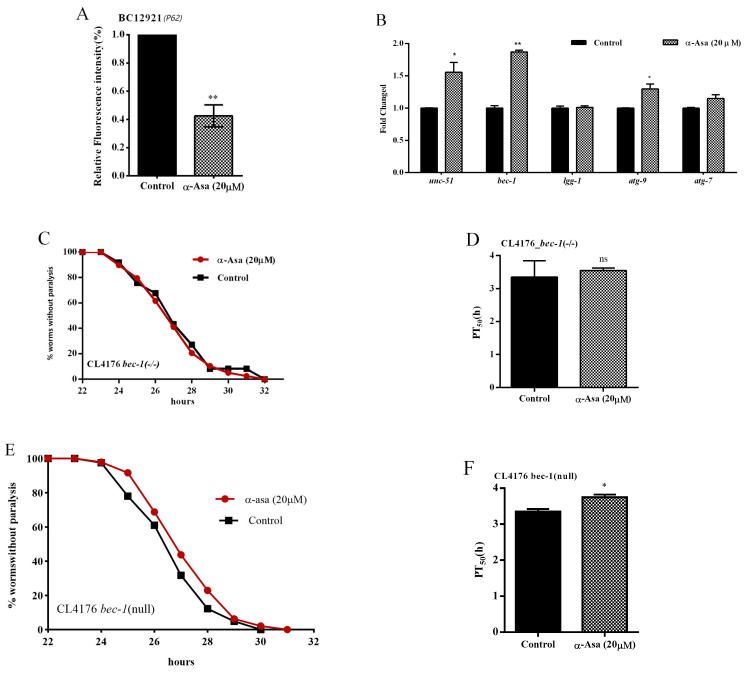
The protective effect of α-asarone against Aβ was related to the autophagy pathway. (**A**) The P62/SQST-1::GFP expression level in BC12921 strains. (**B**) α-Asarone upregulated the transcription levels of *unc-51* (orthologous to human *ulk-1* which participates in autophagy initiation), *bec-1* (key gene to form the phagophore), *atg-9*, *atg-7* (autophagosome formation-associated genes); more than 1000 worms in total. (**C**) α-Asarone did not affect the paralysis of nematodes when *bec-1* was interfered compared with the control group. (**D**) Statistical analysis of PT_50_ in the *bec-1* mutant strain. (**E**) Curves of worms not paralyzed that carry the empty plasmid control (*bec-1* null) strain. (**F**) α-Asarone significantly prolonged the PT_50_ of nematodes in CL4176 *bec-1*-null strain. Each treatment has at least 30 worms, 3 biology replicates for each experiment; difference between mean is shown as mean ± SD, unpaired *t*-test, ns *p* > 0.05, * *p* < 0.05, ** *p* < 0.01. The statistical data is shown in Table S10.

Our phenotype analysis results indicated that α-asarone significantly ameliorated Aβ- and tau-induced damage in *C. elegans*, and the protective effects rely on proteostasis. Autophagy, a lysosome-dependent degradation system, is present in all eukaryotic cells and plays a crucial role in removing misfolded or aggregated proteins intracellularly to maintain proteostasis beyond UPS [34]. Based on this, we sought to determine the interaction between α-asarone treatment and autophagy activity in nematodes. To this end, we employed the well-characterized BC12921 strain in our study, which expresses a p62/SQST-1-GFP fusion protein, allowing us to monitor real-time autophagy changes in vivo [35]. Through a GFP fluorescence assay, we observed a significant decrease in GFP intensity in the BC12921 strain after α-asarone treatment, over 42% compared to the untreated control group (Figure 8A). Subsequently, we examined the expression levels of autophagy-related genes and found that α-asarone treatment significantly increased the mRNA levels of the autophagy membrane formation genes *bec-1*, *unc-51*, and *atg-9* (Figure 8B). Furthermore, in *bec-1* knockdown worms, we did not observe any significant difference in paralysis rates between the α-asarone-treated group and the control group (Figure 8C–F, Table S10). Our results indicated that α-asarone’s protective effects against Aβ-induced damage in nematodes likely rely on stimulating autophagy.

## 4. Discussion

Senile plaques composed of amyloid peptides and neurofibrillary tangles induced by abnormal phosphorylation tau are specific pathological characteristics of Alzheimer’s disease [4]. *Acori tatarinowii* (*A. tatarinowii*) is the most frequently used traditional Chinese medicine to treat dementia. α-Asarone is the active constituent of *A. tatarinowii* and exhibits neuroprotective effects by facilitating angiogenesis and inhibiting neuroinflammation, which has been well demonstrated [36,37]. However, the underlying mechanism of α-asarone in AD treatment has not been fully elaborated.

In this study, we demonstrated that α-asarone not only mitigated Aβ-associated toxicity but also alleviated damage induced by hyperphosphorylated tau in *C. elegans*. Moreover, α-asarone treatment improved phenotypic deficits caused by Aβ aggregation in vivo, as evidenced by enhanced chemotaxis, increased sensitivity to exogenous serotonin, and preserved neuronal integrity. Accumulating evidence suggests that Aβ oligomer accumulation is a key driver of Aβ toxicity, and targeting Aβ degradation pathways represents a promising therapeutic strategy for Alzheimer’s disease [38]. Consistent with this, α-asarone reduced Aβ mRNA and protein levels in Aβ transgenic worms and decreased amyloid deposition in the nematode’s anterior area. Collectively, our findings highlight the neuroprotective potential of α-asarone in counteracting Aβ-induced toxicity by promoting Aβ clearance and reducing its accumulation in vivo.

Aβ aggregation induces neuronal toxicity and generates excessive reactive oxygen species (ROS), leading to increased intercellular oxidative stress [39]. The Keap-Nrf2-ARE signaling pathway plays a crucial role in oxidative stress resistance, yet in some AD patients, Nrf2 protein levels remain relatively low [40]. Our experiments demonstrated that α-asarone reduced Aβ-induced ROS accumulation and significantly upregulated *skn-1*, the *C. elegans* ortholog of human transcription factor *Nrf2*, along with its downstream targets *gst-4* and *gcs-1*. Notably, α-asarone failed to mitigate paralysis in *skn-1* mutant worms, indicating that the neuroprotective effects of α-asarone are SKN-1 dependent.

Defective protein degradation exacerbates Aβ accumulation and disrupts proteostasis [41]. SKN-1/Nrf2 plays a critical role in maintaining proteostasis, not only by regulating protein degradation and maintenance but also by supporting proteasome activity beyond its role in oxidative stress-resistance detoxification [42]. Studies suggested that intracellular Aβ accumulation occurs earlier than extracellular Aβ aggregation in both an AD mouse model and patients [2], with the ubiquitin–proteasome system being the primary mechanism for intracellular protein degradation. Excessive cytoplasmic Aβ impairs proteasome function, disrupts UPS regulation, and further accelerates AD progression [43]. In this study, we observed a significantly decreased proteasome activity in the Aβ-expressing CL4176 strain, further confirming that Aβ accumulation inhibits proteasome function in vivo. However, α-asarone treatment restored proteasome activity by upregulating the expression of proteasomal subunit genes. Interestingly, we also observed that bortezomib treatment caused extreme growth delay at the larval stage. Although bortezomib is widely used as a first-line therapy for many cancers, its toxicity and side effects remain a concern [44]. α-Asarone treatment alleviated this disruption in nematodes, suggesting its potential capability to mitigate bortezomib-induced toxicity and providing insights into combination therapy with proteasome inhibitors.

In the organism, the p62-Keap1-Nrf2 regulatory axis connects the ubiquitin–proteasome system with autophagy, further reinforcing proteostasis [45]. In this study, α-asarone treatment significantly activated autophagy, but the key mediators remain to be identified. Given that α-asarone exerts its protective effects against Aβ-induced damage through SKN-1, the interplay between SKN-1 and autophagy following α-asarone treatment warrants further investigation.

In addition, tau pathology-related neuroinflammation has been closely linked to cognitive dysfunction, and targeting tau has raised as a potential alternative therapeutic strategy for AD [46]. In this study, we observed that α-asarone treatment significantly reduced hyperphosphorylated-tau-induced AD progression, suggesting a potential effect of α-asarone on tau pathology. We hypothesize that α-asarone may ameliorate hyperphosphorylated tau-induced phenotypes through its proteostasis-regulating activity, and further studies are worthwhile to dissect the underlying mechanism. Current Alzheimer’s disease therapeutics primarily offer symptomatic relief by targeting cholinergic and glutamatergic neurotransmission. Despite more than 2700 AD clinical trials conducted over the past two decades, most have failed to yield satisfactory outcomes [47]. The mystery of AD pathophysiology hindered the development of disease-modifying drugs. Given the failure of the single target approach, polypharmacology to maintain protein homeostasis may pave new avenues for AD. Nrf2 is a master regulator in cellular health, influencing various processes, including antioxidative stress, and protein degradation, making it a potential target for neurodegenerative disease treatment. Our results demonstrated that α-asarone mitigates Aβ-induced toxicity in *C. elegans* through SKN-1-mediated proteostasis, implying its therapeutic potential as an Nrf2 activator for AD treatment. However, we did not investigate the protective effect of α-asarone in other AD models, and more investigations should be performed to validate its function in the future.

## Figures and Tables

**Table 1:** *C. elegans* strains used in this study.

Strain	Genotype	Source	Key Characteristics/Use
N2	Wild-type	CGC	Wild-type control
CL4176	smg-1(cc546ts) I; dvIs27 [myo-3p::A-Beta(1-42)::let-851 3′UTR + rol-6(su1006)] X	CGC	Temperature-inducible Aβ expression in body wall muscle
CL2122	dvIs15 [(pPD30.38) unc-54(vector) + (pCL26) mtl-2::GFP]	CGC	Aβ expression control strain
CL2331	dvIs37 [myo-3p::A-Beta(1-42)::GFP + rol-6(su1006)]	CGC	Aβ::GFP fusion protein expression in muscle
CL2006	dvIs2 [pCL12(unc-54::A-Beta(1-42)) + pCL26(mtl-2::GFP)]	CGC	Constitutive Aβ expression in body wall muscle
CL6180	smg-1(cc546ts) I; dvIs19 [gst-4p::GFP] III; skn-1(zu67) IV/nT1 [unc-?(n754) let-?] (IV;V); dvIs27 [myo-3p::A-Beta(1-42)::let-851 3′UTR + rol-6(su1006)] X	CGC	SKN-1 loss-of-function mutant with Aβ background
CL2166	dvIs19 [(pAF15)gst-4p::GFP::NLS] III	CGC	Oxidative stress reporter (GFP under gst-4 promoter)
CL2355	smg-1(cc546ts) I; dvIs50 [pCL12(snb-1::A-Beta(1-42)::3′UTR) + pCL26(mtl-2::GFP)]	CGC	Pan-neuronal Aβ expression
GMC101	dvIs100 [unc-54p::A-Beta(1-42)::unc-54 3′UTR + rol-6(su1006)]	CGC	Aβ aggregate formation model
VH254	pha-1(e2123ts) III; hdEx81 [unc-54p::Tau(4R/2N)-PHP + pha-1(+)]	CGC	Pseudohyperphosphorylated tau expression in muscle
VH255	pha-1(e2123ts) III; hdEx82 [unc-54p::Tau(4R/2N)-WT + pha-1(+)]	CGC	Wild-type human tau expression in muscle
BC12921	dpy-5(e907) I; sIs12716 [hlh-30p::hlh-30::GFP + rol-6(su1006)]	CGC	TFEB/HLH-30 nuclear translocation reporter
EG1285	lin-15B(n744)&lin-15A(n765) X; oxIs12 [unc-47p::GFP + lin-15(+)]	Qi Wang Lab (Peking University)	GABAergic motor neuron marker
PHX3692	smg-1(cc546ts) I; dvIs50 [pCL12(snb-1::A-Beta(1-42)::3′UTR) + pCL26(mtl-2::GFP)]; wgIs3692 [unc-47p::mCherry + rol-6(su1006)]	SunyBiotech Co., Ltd. (Fuzhou, China)	GABAergic neuronal marker in Aβ background

## Data Availability

The datasets used and analyzed during the current study are available from the corresponding author on reasonable request.

## Supplementary Materials

The following supporting information can be downloaded at https://www.mdpi.com/article/10.3390/antiox14101255/s1, Table S1: Statistical analysis of the effects of different concentrations of α-asarone on paralysis in CL4176; Table S2: The thrashing rate in tau transgenic strains; Table S3: α-asarone improved chemotaxis-related learning in CL2355 nematodes; Table S4: α-asarone enhanced 5-HT sensitivity in CL2355 nematodes; Table S5: Data analysis of the relative fluorescence intensity in the anterior area in CL2331 treated with or without 20 μΜ α-asarone; Table S6: The ROS levels of worms after treatment with or without α-asarone; Table S7: The average lifespan of worms under PQ-induced oxidative stress; Table S8: GST-4 protein levels after treatment with α-asarone and control in CL2166 strain; Table S9: Statistical analysis of paralysis in skn-1 mutant CL6180 nematodes; Table S10: Statistical analysis of paralysis in bec-1 RNAi worms treated or untreated with α-asarone; Table S11: The primer sequences of genes.

## Abbreviations

AD, Alzheimer’s disease; Aβ, amyloid peptide; A. tatarinowii, Acorus tatarinowii Schott; APP, amyloid precursor protein; AMC, 7-amido-4-methylcoumarin; *C. elegans*, *Caenorhabditis elegans*; CGC, Caenorhabditis Genetics Center; DMSO, Dimethyl Sulfoxide; DEPC, diethylpyrocarbonate; FUDR, 5-fluoro-2′-deoxyuridine; GABA, gamma-aminobutyric acid; H2DCF-DA, 7′-Dichloro-fluorescein diacetate; IPTG, isopropyl-β-D-1-thiogalactopyranoside; Nrf2, Nuclear Factor E2-Related Factors; NFT, neurofibrillary tangles; NGM, nematode growth medium; PHP, pseudo hyperphosphorylated; qRT-PCR, quantitative real-time PCR; ROS, reactive oxygen species; UPS, ubiquitin–proteasome system; 5-HT, 5-hydroxytryptamine.
