# Supplementary material for: α-Asarone Maintains Protein Homeostasis Through SKN-1-Mediated Proteasome and Autophagy Pathways to Mitigate Aβ-Associated Toxicity in Caenorhabditis elegans
Source: Antioxidants (Basel). 2025 Oct 18;14(10):1255. doi: 10.3390/antiox14101255 (PMC12562124; doi:10.3390/antiox14101255)
Supplement: Supplementary file 1 [file antioxidants-14-01255-s001.zip › antioxidants-3872877-supplementary.pdf]

α-Asarone maintains protein homeostasis through SKN-1-mediated proteasome and autophagy pathways to mitigate Aβ-associated toxicity in *Caenorhabditis elegans*

Congmin Wei#, Xinyan Chen#, Menglu Sun, Jinjin Cao, Dechun Liao, Zhou Cheng*, Hongbing Wang*

Institute for Regenerative Medicine, Shanghai East Hospital, School of Life Sciences and Technology, Tongji University, Shanghai 200092, China

# Equal contribution: These authors contributed equally to this work

*Corresponding authors. Tel./fax: +86 21 65983693 (Hongbing Wang).

E-mail addresses: hbwang@tongji.edu.cn (Hongbing Wang),

Chengzhou@tongji.edu.cn (Zhou Cheng)

Table S1. Statistical analysis of the effects of different concentrations of α-asarone on paralysis in CL4176

Treatment	Number of worms	PT_{50} (h)	Percentage changed (%)	*p* value
Control	156	6.00 ± 0.20	/	/
α-Asarone (2 μM)	90	6.93 ± 0.51	15.50	0.1185
α-Asarone (20 μM)	117	9.73 ± 0.32	62.17	0.0001***
α-Asarone(200 μM)	117	7.93 ± 0.78	32.17	0.0402*

Table S2. The thrashing rate in tau transgenic strains

Strains and treatments	Concentration (μM)	Thrashing rate/10 s	Number of worms
VH-255 Control	0	6.061 ± 0.0606	90
VH-255 α-asarone	20	6.155 ± 0.3881	90
VH-254 Control	0	3.198 ± 0.1016	90
VH-254 α-asarone	20	5.705 ± 0.0619***	90

Table S3. α-asarone improved chemotaxis-related learning in CL2355 nematodes

Strains	Treatments	Concentration (μM)	Number of worms	CI
CL2355	Control	0	184	-0.08 ± 0.02
	α-asarone	20	158	0.18 ± 0.03**
CL2122	Control	0	257	0.36 ± 0.01****

Table S4. α-asarone enhanced 5-HT sensitivity in CL2355 nematodes

Strains	Treatments	Concentration (μM)	Number of worms	Active worms (%)
CL2355	Control	0	130	23.75 ± 0.49
	α-asarone	20	140	43.38 ± 1.06**
CL2122	Control	0	180	60.55 ± 4.00
	α-asarone	20	180	60.55 ± 4.34ns

Table S5. Data analysis of the relative fluorescence intensity in the anterior area in CL2331 treated with or without 20 μM α-asarone

Treatment	Number of worms	Percentage of worms of deposited on the anterior area (%)	*p* value
Control	91	36.96 ± 0.45	/
α-asarone (20 μM)	96	26.39 ± 1.12	<0.0001(****)

Table S6. The ROS levels of worms after treatment with or without α-asarone

Treatment	Number of worms	ROS level (%)	Percentage changed (%)	*p* value
Control	270	100	-	/
Resveratrol	270	81.28 ± 3.31	18.72	0.0048**
α-asarone (20 μM)	270	86.79 ± 1.64	13.21	0.0013**

Table S7. The average lifespan of worms under PQ induced oxidative stress

Treatment	Number of worms	Average lifespan (h)	Percentage changed (%)	*p* value
Control	116	8.43 ± 0.31	-	/
α-asarone (20 μM)	117	10.24 ± 0.34	21.47	0.0357*

Table S8. GST-4 protein levels after treatment with α-asarone and control in CL2166 strain

Treatment	Number of worms	Relative fluorescence intensity (%)	*p* value
Control	90	100	
α-asarone	90	120.5 ± 1.03	0.0025**

Table S9. Statistical analysis of paralysis in *skn-1* mutant CL6180 nematodes

Treatment	Number of worms	Average lifespan(h)	*p* value
Control	66	25.59 ± 0.17	-
α-asarone	75	25.21 ± 0.49	0.54

TableS10. Statistical analysis of paralysis in *bec-1* RNAi worms treated or untreated with α-asarone

Treatment	Number of worms	PT_{50}(h)	*p* value
Control_CL4176_*bec-1*(-/-)	72	3.35 ± 0.35	-
α-Asarone_CL4176_*bec-1*(-/-)	72	3.55 ± 0.05	0.63
Control_L4440(vector)	125	3.35 ± 0.05	-
α-asarone_L440(vector)	139	3.75 ± 0.05	0.03

Table S11. The primer sequences of genes

gene	forward primer sequence	reverse primer sequence
actin-1	CCAGAAGAGCACCCAGTC	TGATGTCACGGACGATTT
Aβ	CAGAATTCCGACATGACTCAGG ATATGAAG	CCCACCATGAGTCCAATGATTGC
skn-1	CACGCCGTCAGCGAAGTA	ATGCTCGGTGAGTATTGG
gst-4	ACCAGCCCGTGATGATTTCT	ATCCTTTCTTGTTGCCACGT
unc-51	CGCCGGTGGTTCAGCGGATT	TATCCTGGGTGTCGGCGGGG
lgg-1	GCCGAAGGAGACAAGATCCG	GGTCCTGGTAGAGTTGTCCC
bec-1	TTGAAGAAATTGTTGGCTGAGG	AACTTCTGCATATTGACGTTCG
atg-9	ATCTAAAACACGAAATCGAGCG	CTCGTGATGTTTGTACTCCTCT
atg-7	CCAAAAGCTGTGGGATGGGA	GCGTTCCAGCACCAAGAATG
pbs-1	TCTCATCGCTATGGAGTACAAC	GCACACAACCATATTATCGGTG
pbs-2	GCCCACATCTTTATATGTGCTC	TCTCGGCTTCATCTTTCGT
pbs-5	CAAGTACTGCACTTTGTACGAG	ATTTGTGGTCCCTTCTTATCGT
pas-1	AGGACCAGAAGTTTATCGTGTT	AATGAAGTCTGAAGAGCCTCAA
pas-2	CATTGCTCTTCCAATGTGATCC	GAGCAGTGTGAATTCCATCATC
pas-3	AATGAAAAGCAGAGATACACGC	CTCCTGTTTCAACAAGGTTACG
pas-4	AAATTGGTTGTAAAGTCGCTGG	GCTGCAGCTCATCATTAACTTT
pas-5	GATCGAGAAAATCAGCAAGGTC	GTGAACCAGAAATTTTGAGCCT
pas-6	CTGATTGCCGGATATGACAAAG	GATCTTGCTCCGATTGAAGTTC
pas-7	GTGGCAAGAACGGAGTTGTG	AAACGGACGACTAATGCCGA
rpn-10	GCGTTGTTGCAGTCAAGTGT	TGCTCTTCAGTCATCGCTCC
rpn-6	GATGGAGATATTAAGGTTGGAC	TCATTATAGAGGCGAACAAGAC
rpn-11	GTCATTTGCAGAAGCCGAGC	TCCTTGTTCTGTTCACCGCA
rpt-2	AACACCAATGGCAGTCGGAT	GCTCACGGACCATCTTTGGA
rpt-5	CATCCTCCAAAGGGTGTGCT	ACCAGAACGAAGAAGAGCGG
rpt-6	AAATGTGCGTCGTCTGCAAG	TGACTGGCAGCTCGATAACC
bec-1(RNAi)	GCTCTAGAATGACGACCCAACGAAGCCA	GGGGTACCCTAAATAGGCGATCTGAGAG
